# Genomic and Conventional Inbreeding Coefficient Estimation Using Different Estimator Models in Korean Duroc, Landrace, and Yorkshire Breeds Using 70K Porcine SNP BeadChip

**DOI:** 10.3390/ani14172621

**Published:** 2024-09-09

**Authors:** Kefala Taye Mekonnen, Dong-Hui Lee, Young-Gyu Cho, Ah-Yeong Son, Kang-Seok Seo

**Affiliations:** 1 Department of Animal Science and Technology, Sunchon National University, Suncheon 57922, Republic of Korea; kefala.taye@arsiun.edu.et (K.T.M.);; 2 Department of Animal Science, College of Agriculture and Environmental Science, Arsi University, Asella P.O. Box 193, Ethiopia

**Keywords:** correlation, genomics, inbreeding coefficient, pedigree, pig breed, run of homozygosity

## Abstract

**Simple Summary:**

Inbreeding increases homozygosity, often leading to the co-expression of recessive deleterious alleles and a reduction in genetic diversity. Traditional methods involve pedigree-based analysis, but high-density arrays have enabled precise estimations using genomic markers. A study comparing genomic inbreeding coefficients from DNA marker data with conventional coefficients from pedigree information in Korean commercial pig breeds reveals differences in inbreeding levels and trends among breeds. The finding highlights differences in inbreeding levels and trends among breeds, emphasizing the importance of using both genomic and conventional methods for a comprehensive understanding of inbreeding, which is crucial for effective breeding programs and maintaining genetic diversity.

**Abstract:**

The purpose of this study was to estimate the homozygosity distribution and compute genomic and conventional inbreeding coefficients in three genetically diverse pig breed populations. The genomic and pedigree data of Duroc (1586), Landrace (2256), and Yorkshire (3646) were analyzed. We estimated and compared various genomic and pedigree inbreeding coefficients using different models and approaches. A total of 709,384 ROH segments in Duroc, 816,898 in Landrace, and 1,401,781 in Yorkshire, with average lengths of 53.59 Mb, 56.21 Mb, and 53.46 Mb, respectively, were identified. Relatively, the Yorkshire breed had the shortest ROH segments, whereas the Landrace breed had the longest mean ROH segments. *Sus scrofa* chromosome 1 (SSC1) had the highest chromosomal coverage by ROH across all breeds. Across breeds, an absolute correlation (1.0) was seen between F_ROH_ total and F_ROH1–2Mb_, showing that short ROH were the primary contributors to overall F_ROH_ values. The overall association between genomic and conventional inbreeding was weak, with values ranging from 0.058 to 0.140. In contrast, total genomic inbreeding (F_ROH_) and ROH classes showed a strong association, ranging from 0.663 to 1.00, across the genotypes. The results of genomic and conventional inbreeding estimates improve our understanding of the genetic diversity among genotypes.

## 1. Introduction

Inbreeding occurs when related individuals reproduce, resulting in offspring with reduced genetic variation, which is unavoidable for closed and selected populations. As inbreeding increases, genetic variation decreases, leading to depression [1]. The individual inbreeding coefficient (F) measures the fraction of an individual genome that is homozygous due to common ancestry. The average of all individual F values indicates the overall amount of inbreeding within a population [2]. Conventionally, researchers and breeders used Wright’s path coefficient techniques [3] to estimate inbreeding coefficients from pedigree information. However, the advent of modern technology has led to the emergence of alternative methods for estimating inbreeding coefficients. One such approach involves using molecular markers to estimate inbreeding when pedigrees are unavailable. This is accomplished by analyzing the disparity between the observed and expected multi-locus heterozygosity [4].

The recent creation of genome-wide SNP bead chips with a high density has sparked new interest in using molecular data to figure out individual inbreeding coefficients [5]. SNPs allow us to estimate inbreeding levels by analyzing the variance of genotype values [6]. Alternatively, Yang et al. [7] suggest using a combination of the variance of genotype values and the levels of homozygosity. Elevated levels of inbreeding increase the likelihood of homozygous deleterious recessives, widely regarded as the primary factor contributing to inbreeding depression. In order to mitigate the adverse consequences of inbreeding, it is imperative to precisely and delicately assess the degree of consanguinity. Using runs of homozygosity (ROH) to estimate the genomic inbreeding coefficients (F_ROH_) is currently the best and most accurate way to figure out the effects of genetic inbreeding and how much genetic material is shared across the whole genome [8,9]. According to whole-genome sequencing, ROH is a contiguous chromosomal region in which an individual inherits identical haplotypes from both parents [10]. A pair of identical (homologous) haplotypes derived from a common ancestor creates these long tracts of homozygous genotypes [10,11]. The haplotypes inherited from recent common ancestors tended to be longer, whereas those inherited from distant ancestors tended to be shorter [11]. Animal genetics extensively uses ROH analysis to revolutionize the conventional method of inbreeding effect estimation, by which we usually figure out how inbreeding affects animals [5,12,13].

ROHs can accurately predict alleles at loci that are identical by descent (IBD). It is also commonly used by geneticists and researchers to evaluate the autozygosity levels, which represent the homozygous presence of IBD alleles in human and livestock populations [11,14]. They provide a valuable tool for estimating the inbreeding coefficient levels and assessing the genetic structure of economically relevant attributes and relationships across the entire genome of livestock species [9,15,16]. In animal genetics, the location of recurrent diseases, the history of the population, and the level of consanguinity all affect the frequency of homozygous segments [17]. Genetic drift, population bottlenecks, mating with the same family, and both natural and artificial selection pressure can all significantly contribute to an increase in ROHs within a population [11,14]. ROH analysis is beneficial for devising mating plans aimed at minimizing livestock inbreeding because it identifies shared genomic segments transmitted from an individual to their offspring, allowing for adjustments in breeding strategies to maintain genetic diversity and mitigate the negative effects of inbreeding [18,19].

Furthermore, ROH analysis was employed to investigate the relationship between genotypes and phenotypes and to pinpoint regions linked to economically important traits in livestock [19,20,21]. Frequently, ROH patterns in livestock align with their breeding history or lineage. Positive selection or long-term breeding programs tend to exhibit high-frequency ROH in genomic regions [19]. These high-frequency ROH regions indicate the influence of both natural and artificial selection on specific genome segments during animal breeding [21]. The location of ROH indicates the genomic segments that have undergone selective pressures [22,23].

Previous research has investigated the association between homozygosity and complex diseases in humans, in which the presence of autozygosity or homozygosity for deleterious recessive alleles can effectively reflect the underlying causes of recessive diseases [17,24,25,26,27]. Advancements in high-density genotyping chips and sequencing technologies have made it easier to investigate the study of homozygosity in various populations [17]. The advancements, along with enhanced techniques for identifying runs of homozygosity (ROH), have led to a growing curiosity in investigating the origins and trends of homozygosity in recent times [28].

Therefore, the current study focused on the Korean Duroc, Landrace, and Yorkshire breeds, which are the major commercial pork breeds in Korea. These breeds have developed over time through selection and crossbreeding. The Landrace breed is a large white pig that originated in Denmark; the Yorkshire breed comes from England; and the Duroc breed originates from the United States. However, to our knowledge, no studies have combined genomics based on runs of homozygosity (ROH) with traditional inbreeding coefficient estimation using various estimator models in the Korean Duroc, Landrace, and Yorkshire breeds. This study’s goal was to describe the distribution of homozygosity (ROH), use this information to estimate genomic inbreeding coefficients, and compute and compare with inbreeding coefficients based on pedigree information. We used a variety of genomic inbreeding estimator methods to obtain comprehensive and precise estimates of inbreeding coefficients. A number of estimators were used, including ROH (F_ROH_), F_HOM_ (the number of homozygous genotypes seen compared to what was expected), F_HAT1_ (the variation of additive genotype values), F_HAT2_ (excess homozygosity), F_HAT3_ (the correlation between uniting gametes), and F_PED_ (pedigree-based inbreeding coefficients). We also computed the correlation between the genomic and pedigree-based inbreeding coefficients by comparing the inbreeding coefficients generated by different estimator approaches. These comparisons and analyses are crucial for assessing inbreeding levels and guiding appropriate breeding strategies on pig breeding farms.

## 2. Materials and Methods

### 2.1. Animal and Phenotypic Data

Table 1 shows the total number of animals used to estimate the pedigree and genomic inbreeding coefficients across the three breeds. Data from three genetically distinct swine breeds, born from 2010 to 2022, were used to estimate conventional inbreeding coefficients. The dataset consisted of 76,987, 155,628, and 409,018 pedigree records for the Duroc, Landrace, and Yorkshire pig populations, respectively, with pedigree data traced back to 15 generations. The analysis of each individual pedigree inbreeding coefficient (F_PED_) included only animals with complete information on both parents’ lineages. Consequently, the analyzed pedigree data contained information about animals with both sires and dams (99.21%), animals with only sires (0.07%), and animals with no recorded parental information (0.71%). 

### 2.2. Genotyping and Quality Control

For ROH analysis, genomic data from 1586 Duroc, 2256 Landrace pigs, and 3646 Yorkshire breeds were used. The blood for DNA analysis was collected from animals kept in five GGP commercial breeding farms. All the three different genotypes were bred using artificial insemination. DNA was extracted from blood samples, and genotyping was performed using Porcine70KSNP BeadChips (Illumina, San Diego, CA, USA). The PLINK software (v1.90) was used to keep SNPs with MAF > 0.05, mind 0.1, SNP call rate >95%, individual call rate >95%, and HWE > 1 × 10^−6^ before conducting ROH detection and classifications for quality control. MAF was used to filter SNPs during quality control. Reducing potential genotyping errors and ensuring a higher MAF in the dataset could improve ROH detection’s reliability. On the other hand, the inclusion of HWE helps improve the accuracy of ROH detection, where deviations from HWE can lead to misinterpretation of ROH patterns. Principal component analysis (PCA) was applied to differentiate genetic clustering between the three breeds.

### 2.3. Detection and Classification of ROH

We used a meticulous methodology to identify patterns of runs of homozygosity (ROH) in the genomes of three distinct pig breed populations, using the R program “*detectRUNS*” to ascertain the frequency of each single nucleotide polymorphism (SNP) within the Regions of Homozygosity (ROHs). The number of SNPs across the entire population was counted to determine the frequency of SNPs within ROH and identified SNPs with occurrence frequencies in the top 1% as likely regions of homozygosity islands [17,29,30]. We utilized a window size of 15 SNPs and a scanning threshold of 0.05 to identify ROH throughout the genome. Each ROH segment comprised a minimum of 20 SNPs, excluding heterozygosity, and allowed for a maximum of one opposite window and one maximum window, with gaps of up to 10^6^ bp permissible within the ROH segments. To ensure accurate ROH detection, we set the minimum length of the ROH segments at 250,000 base pairs and the minimum SNP density at one SNP per 1000 base pairs (SNP/kbps). We carefully selected these parameters to ensure robust ROH detection, minimize false positives, and maintain sensitivity in detecting meaningful homozygosity patterns in the genomic data. To generate a summary output for the identified ROHs, we computed a ROH summary using a sliding window methodology. We specifically defined the SNP class within runs by setting the parameter class to two, and we included SNPs found within the identified runs in the resultant summary. This process generates information about the detected runs across the examined genomic regions. According to their length, we classified the identified ROHs into five different classes: 1–2, 2–4, 4–8, 8–16, and >16 Mb, following suggestions in previous studies involving various cattle breeds and buffaloes [13,20,31,32]. Subsequently, we used R (http://www.R-project.org/, accessed on 25 April 2024) to calculate, summarize, and graphically visualize the frequency, average length (Mb), percentage, and genome coverage percentage for each class of ROH across all individuals in each of the three breeds.

### 2.4. Estimation of Pedigree Inbreeding Coefficients (F_PED_)

Pedigree-based inbreeding coefficients (F_PED_) were calculated by tracking ancestral relationships among individuals and estimating the inbreeding coefficient based on known pedigree information. The pedigree R package [33,34] was used to perform the analysis, and the conventional inbreeding coefficient (f) was computed without taking genetic groups into account, with missing ancestors assigned a value of zero (0). We also computed the equivalent complete generations (ECG) to assess the depth of a population and determine the number of known ancestors for the three genotypes, which we then compared with genomic inbreeding, as proposed by Maignel et al. [35].

### 2.5. Estimation of Genomic Inbreeding Coefficient

We estimated the genomic inbreeding coefficient using the ROH in the genome and excess homozygosity (HOM) based on the observed versus expected number of homozygous genotypes to identify individuals with a high degree of relatedness. To reduce the incidence of random ROH, the minimum number of SNPs that formed an ROH (*l*) was determined using the method proposed by Lencz et al. [24] and adapted by Nishio et al. [36] and Purfield et al. [12], as follows:

(1) $$l=\frac{\log_{e}\frac{\alpha }{n_{s}\cdot n_{i}}}{\log_{e}\left(1-het\right)}$$

where $n_{s}$ is the number of SNPs per individual, $n_{i}$ is the number of individuals, α is the percentage of false positive ROH, and het is the mean SNP heterozygosity across all SNPs. Thus, to compare with the conventional method for inbreeding coefficients, we used different estimator models based on genomic information: F_ROH_, F_ROH1–2_, F_ROH2–4_, F_ROH4–8_, F_ROH8–16_, F_ROH>16_, F_HOM_, F_HAT1_, F_HAT2_, and F_HAT3_. To detect sliding ROH, we used PLINK v1.90 [37], GCTA software v1.94.1 [38], and “*detectRUNS*” in the R package [33]. SAS 2012 ver. 9.4 software was used to statistically summarize the results and graphically visualize them using R software ver.4.3.3.

The first method of genomic inbreeding coefficient estimation depends on ROH, denoted as F_ROH_, which is described as the length of the genome present in ROH divided by the total length of the genome covered by single nucleotide polymorphisms [39,40]:

(2) $$F_{ROH}=\frac{\sum L_{ROH}}{L_{genome}}$$

where $\sum L_{ROH}$ is the sum of the lengths of all ROH detected in an individual, and $L_{genome}$ is the total length of the genome size of autosomes covered by markers, set at 2,866,838,672 base pairs (bp) based on the sliding Run map that was used. We calculated F_ROH_ for each animal in each breed population group based on their genomic length (F_ROH1–2Mb_, F_ROH2–4Mb_, F_ROH4–8Mb_, F_ROH8–16Mb_, and F_ROH>16Mb_) and classified them into five distinct classes on the basis of their genomic lengths: 1–2 Mb, 2–4 Mb, 4–8 Mb, 8–16 Mb, and >16 Mb, respectively. The second method of genomic inbreeding, which involves comparing the observed and expected counts of autosomal homozygous genotypes (F_HOM_), was calculated using the flag *—het* in PLINKv1.90b7.2 [37] for each individual as follows:

(3) $$F_{HOM}=\frac{O_{HOM}-E_{HOM}}{L-E_{HOM}}$$

where L is the number of genotyped autosomal SNPs, $E_{HOM}$ is the number of expected and $O_{HOM}$ is the number of observed homozygous SNP under the Hardy-Weinberg principle. The estimator is based on SNP-by-SNP analysis and the representative nomenclature of the estimator $\left(F_{HOM}\right)$, as found in the literature [41,42] and $\left(F_{H}\right)$ [43]. The third method of genomic inbreeding coefficient estimators $\left(F_{HAT},F_{HAT2}\;and\;F_{HAT3}\right)$ have been primarily developed in the GCTA software v1.94.1 [38] and they all represent a SNP-by-SNP analysis and can be obtained simultaneously with the flag —*ibc* in PLINK v1.9.

The $F_{HAT1}$ estimator of genomic inbreeding coefficient is the usual variance-standardized relationship minus 1 and is calculated using the following formula:

(4) $$F_{HAT1}=\frac{1}{n}\sum_{m=1}^{n}\frac{{\left(X_{m}-{2p}_{m}\right)}^{2}}{{2p}_{m}q_{m}}$$

$F_{HAT2}$ is analogous to the $F_{HOM}$ estimator measuring the excess of homozygosity and the difference between $F_{HOM}$ and $F_{HAT2}$ is that $F_{HOM}$ is a ratio of sums, whereas $F_{HAT2}$ is a sum of ratios [44] and is calculated using the following formula:

(5) $$F_{HAT2}=\left(1-\frac{1}{n}\sum_{m=1}^{n}\frac{{\left(x_{m}-{2p}_{m}\right)}^{2}}{{2p}_{m}q_{m}}\right)$$

The $F_{HAT3}$ estimator is Wright’s actual definition of genomic inbreeding coefficient (correlation between uniting gametes) [3,7,45] and is estimated using the following formula:

(6) $$F_{HAT3}=\frac{1}{n}\sum_{m=1}^{n}\frac{X_{m}^{2}-\left(1+{2p}_{m}\right)X_{m}+{2p}_{m}^{2}}{{2p}_{m}q_{m}}$$

where X represents the matrix of (n×m) genotypes, coded according to the number of copies of the defined reference allele and, p and q represent the frequencies of the reference and alternative alleles, respectively, summarized across m SNP of k individuals. We also calculated the Pearson’s correlation coefficient between F_ROH_ and other genomic inbreeding estimates, along with the genomic and pedigree inbreeding coefficients for each breed group.

## 3. Results and Discussion

Using pedigree and genomic data, this study identified the level of inbreeding and the accuracy of estimators in three Korean breeds. We investigated the genomic distribution of ROH segments in three genetically diverse Korean breeds. We estimated the genomic inbreeding coefficients utilizing various approaches, which involve F_ROH_, F_HOM_, F_HAT1_, F_HAT2,_ F_HAT3_, and the pedigree-based inbreeding coefficient (F_PED_). Additionally, we compared the correlations between estimates obtained from conventional and genomic inbreeding coefficient methods. Furthermore, we analyzed the trends in inbreeding coefficients based on different ROH class sizes within the Duroc, Landrace, and Yorkshire Korean breeds.

### 3.1. Equivalent Complete Generations (ECG)

Equivalent complete generation (ECG) is a measure of population depth that indicates the number of generations of known ancestors [35]. The results in Table 2 summarize the descriptive statistics for equivalent complete generations (ECG) in genotyped breeds based on pedigree information. The mean ECG values for the Duroc, Landrace, and Yorkshire breeds were 14.89, 14.94, and 14.92 generations, respectively, indicating similar levels of known ancestral generations (Table 2). The pedigree data showed that the mean ECG value for all three genotyped breeds was 14.89 generations, with a minimum of 1 generation and a maximum of 15 generations (Table 2), serving as an ECG for the genomic inbreeding coefficient based on run of homozygosity (F_ROH_).

### 3.2. Genomic Distribution of Runs of Homozygosity

We investigated the genomic distribution of ROH segments in Korean Duroc, Landrace, and Yorkshire breeds. The total number of ROH segments identified was 709,384, 816,898, and 1,401,781 in the 1586 Duroc, 2256 Landrace, and 3646 Yorkshire breeds, respectively. The mean lengths of the ROH segments were 53.59 Mb, 56.21 Mb, and 53.46 Mb for the Duroc, Landrace, and Yorkshire breeds, respectively. Table 3 summarizes the descriptive statistics of ROH and the length of ROH in the Duroc, Landrace, and Yorkshire breeds, categorized into five ROH segments: 1–2 Mb, 2–4 Mb, 4–8 Mb, 8–16 Mb, and greater than 16 Mb. In the Duroc breed, shorter segments (1–2 Mb) constitute the majority of the total runs of homozygosity (ROH), accounting for 65.18% of the ROH. ROH segments of 2–4 Mb, 4–8 Mb, 8–16 Mb, and >16 Mb contribute 20.20%, 9.59%, 3.50%, and 1.53%, respectively, to the total ROH. Shorter segments (1–2 Mb) are the predominant percentage of the total ROH in Landrace breeds, accounting for 70.58%. The segments with sizes ranging from 2 to 4 Mb, 4 to 8 Mb, 8 to 16 Mb, and greater than 16 Mb contribute 18.35%, 7.23%, 2.54%, and 1.31%, respectively. Similarly, shorter ROH segments (1–2 Mb) constitute a substantial proportion of overall ROH in the Yorkshire breed, accounting for 70.19%. ROH segments of 2–4 Mb, 4–8 Mb, 8–16 Mb, and >16 Mb follow, contributing 18.89%, 7.09%, 2.56%, and 1.27%, respectively, to the overall ROH. An ancestral inbreeding history reflects a short ROH, while recent inbreeding typically forms long ROH segments [46]. 

The Yorkshire breed has the highest number of short ROH segments among the three breeds, indicating a higher level of recent inbreeding or a larger effective population size. In contrast, the Landrace breed had the longest mean ROH segment length, particularly in the >16 Mb category, which could indicate ancient inbreeding events or a smaller historical population size. The Duroc breed, on the other hand, falls between these two extremes, implying that ancient and recent inbreeding occurrences may have influenced this population. Nonetheless, recent inbreeding and selection forces have had the greatest impact on the genome. Other researchers have reported similar homozygosity (ROH) distributions in other pigs [47,48], as well as in livestock species such as sheep [49] and cattle [31].

**Figure 1:**
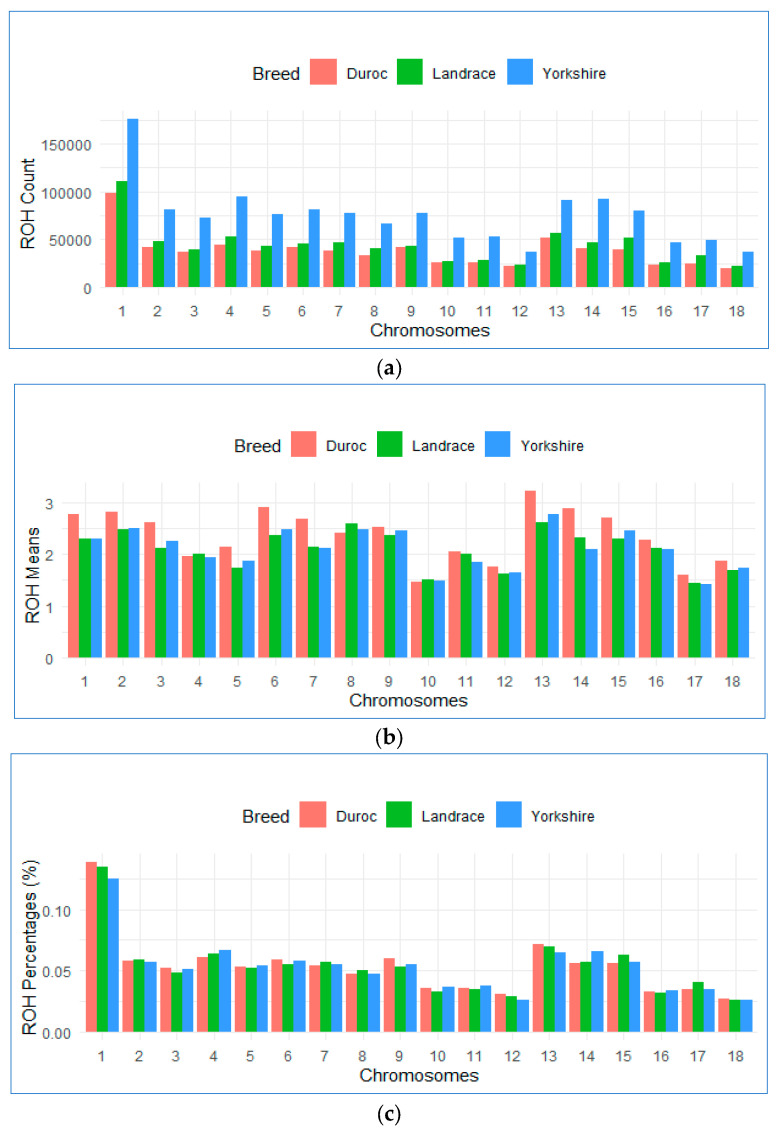
Bar plot for autosomal chromosome across the three breeds. (**a**) number of ROH counts per chromosome; (**b**) means of the ROH count for each chromosome (**c**) percentage of ROH for each autosomal chromosome.

Figure 1a–c and Table 4 depicted the distribution of the total number and means of ROHs in each autosomal chromosome as well as the percentage coverage per chromosome for the three genetically distinct genotypes.

**Figure 2:**
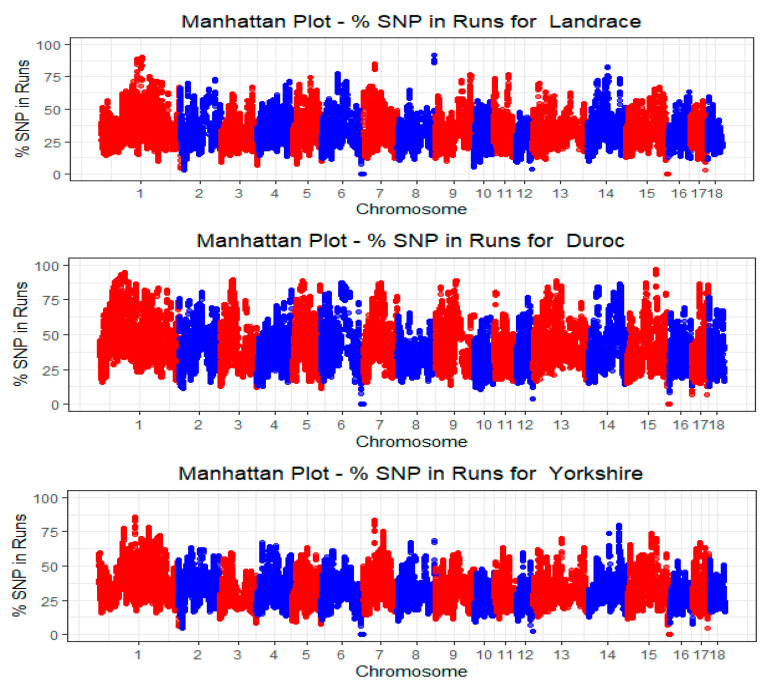
Manhattan plot showing the occurrence frequency of SNPs within ROH for Duroc, Landrace, and Yorkshire pig breeds, identified using a sliding-window run detection approach on autosomal chromosomes with a significance threshold set at 0.05 for ROH delineation.

Across all three breeds, SSC1 consistently showed the highest number of ROHs. Duroc has a total count of 97,815 ROHs (covering 14.31% of the chromosome). Landrace breeds had 110,018 ROHs (14.05% coverage), and Yorkshire breeds had the highest count of 175,188 ROHs (13.12% coverage), suggesting that SSC1 is the largest chromosome and harbors more ROH and markers across the pig genome. The Manhattan plot analysis in Figure 2 also reveals that the Duroc, Landrace, and Yorkshire breeds all had a higher percentage of homozygosity (ROH) in SSC1. Conversely, SSC18 had the smallest number of ROHs and percentage coverage among all breeds: Duroc (19,252), Landrace (21,610), and Yorkshire (36,265), indicating a smaller number of markers across the genome.

Our finding indicates that positive selection may have influenced chromosomes with high ROH coverage across breeds, resulting to the accumulation of beneficial alleles on the chromosome. Previous studies on different pig genomes revealed the highest number of ROH on SSC1, presumably because SSC1 is the largest chromosome in the porcine genome and contains a greater number of markers compared to other chromosomes [46,48,50], which is consistent with our results.

**Figure 3:**
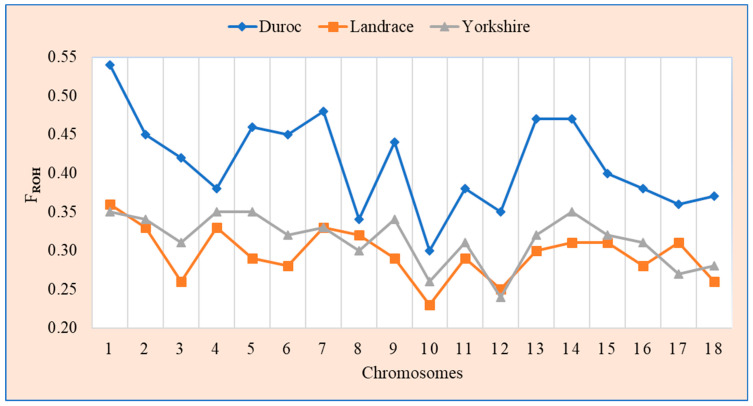
Line plot of chromosome−wide inbreeding coefficient (F_ROH_) for each autosomal chromosome across three distinct pig breeds. The F_ROH_ statistic measures inbreeding by determining the proportion of the genome that is homozygous owing to the recent common ancestry.

**Figure 4:**
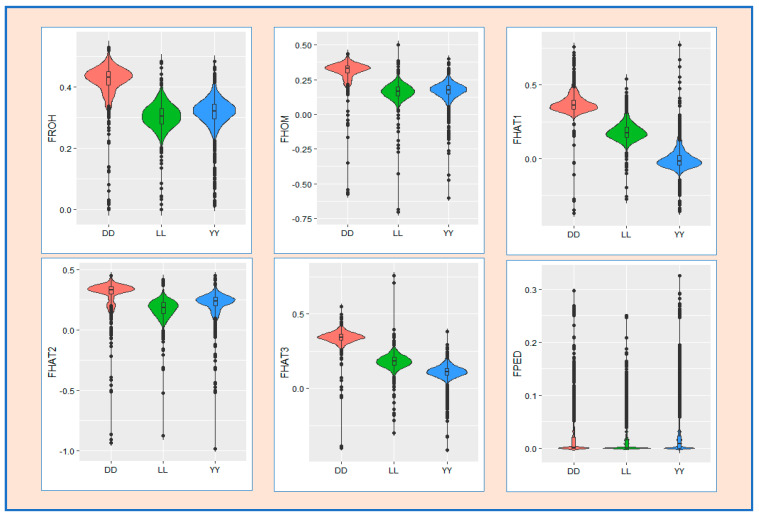
A violin plot of distribution of inbreeding coefficients. F_ROH_: based on ROH; F_HOM_: based on the observed versus expected number of homozygous genotypes; F_HAT1_: based on the variance of additive genotype values; F_HAT2_: based on excess homozygosity; F_HAT3_: based on the correlation between uniting gametes; F_PED_: pedigree−based inbreeding coefficient. DD: Duroc; LL: Landrace; and YY: Yorkshire.

Figure 3 presents a line plot of the chromosome-wide inbreeding coefficient based on ROH (F_ROH_) for 18 autosomal chromosomes across the three distinct genotypes. The results indicate that the Duroc breed has higher values for F_ROH_ at each chromosome level than the Landrace and Yorkshire breeds do. The violin plot of the distribution of genomic and pedigree inbreeding coefficients presented in Figure 4 indicates that the Duroc breed had higher values for all genomic and pedigree inbreeding coefficients than the Landrace and Yorkshire breeds. 

### 3.3. Genomic and Pedigree Inbreeding Coefficient

Table 5 presents the genomic inbreeding coefficients based on ROH across different length classes (F_ROH1–2Mb_, F_ROH2–4Mb_, F_ROH4–8Mb,_ F_ROH8–16Mb,_ and F_ROH>16Mb_) in Duroc, Landrace, and Yorkshire breeds. The results showed that throughout the five classes, the genetic inbreeding coefficient estimated using ROH ranged between 0.42 and 0.08, 0.30 and 0.06, and 0.32 and 0.06 for the Duroc, Landrace, and Yorkshire breeds, respectively. The shortest ROH class (F_ROH1–2Mb_), the genomic inbreeding of Duroc (0.42), was relatively higher than that of the Landrace (0.30) and Yorkshire (0.32) breeds, indicating a greater degree of homozygosity for the Duroc breed population; hence, a higher level of genomic inbreeding. For the intermediate class of ROH (F_ROH4–8Mb_), the genomic inbreeding of the Duroc breed decreased; however, it was higher than that of the Landrace and Yorkshire breeds. When considering the longer ROH classes (F_ROH8–16Mb_ and F_ROH>16Mb_), all three breeds show a decrease in mean F_ROH_ values. Duroc pigs have a mean F_ROH_ of 0.14 and 0.08, respectively, while Landrace and Yorkshire pigs have genomic inbreeding coefficients of 0.09 and 0.06 for the same classes. This suggests that as the ROH length increases, the level of genomic inbreeding based on the ROH class decreases for all breeds, with Duroc consistently displaying higher levels of inbreeding across all classes. A comparison of the genomic inbreeding estimated in this way overestimated the inbreeding coefficient when compared directly with the conventional (pedigree) inbreeding coefficient. However, Maignel et al. [35] suggest using the age of inbreeding with the equivalent complete generation (ECG) of pedigrees for better comparisons. The results in Table 6 also indicate five different genomic (F_ROH_, F_HOM_, F_HAT1_, F_HAT2_, and F_HAT3_) and pedigree inbreeding coefficient (F_PED_) estimates for the three breeds, which indicated a higher inbreeding coefficient for the Duroc breed than for the other two breeds.

The age of inbreeding can be characterized as the degree of genetic relatedness between individuals, which is directly proportional to the length of the ROH [51,52]. Under the assumption that 1 cm equals 1 Mb, the inbreeding coefficient based on runs of homozygosity (F_ROH_) can estimate ancestral populations, with values corresponding to ancestry from 50 generations ago for F_ROH1–2Mb_, 20 generations ago for F_ROH2–4Mb_, 12.5 generations ago for F_ROH4–8Mb_, 6 generations ago for F_ROH8–16Mb_, and 3 generations ago for F_ROH>16Mb_ [51]. Based on the reference point, we calculated the average pedigree depth of 14.92 generations ago for all breeds, and the F_PED_ estimate should be approximately comparable with the genomic inbreeding coefficient of the ROH segment class of 4–8 Mb (F_ROH4–8Mb_). Hence, the genomic-based inbreeding coefficients (F_ROH4–8Mb_) estimated using ROH for Duroc (0.14), Landrace (0.09), and Yorkshire (0.09) were higher than those estimated based on the pedigree for Duroc (0.02), Landrace (0.01), and Yorkshire (0.02), respectively. Furthermore, the F_ROH4–8Mb_ values were higher for all three breeds across the genome than the pedigree-based coefficients (F_PED_), suggesting that genomic data may capture more recent inbreeding events that are not recorded in the pedigree or that the pedigree data are incomplete or inaccurate. The discrepancy between these two estimates can be ascribed to the reality that F_PED_ considers that the complete genome does not experience selection or recombination events [53]. Therefore, they fail to consider the potential bias resulting from these events [54]. Because an incomplete pedigree does not account for distant inbreeding, one can only compare estimates derived by F_PED_ to F_ROH_ calculated across extensive runs of homozygosity [55]. Furthermore, it is crucial to emphasize that the calculation of pedigree resemblance relies on statistical predictions of the likely fraction of identical-by-descent (IBD) genomic segments, whereas genotype-based estimations provide a more accurate measure of relatedness between individuals [56].

### 3.4. Genomic and Pedigree Inbreeding Coefficients Correlations

**Figure 5:**
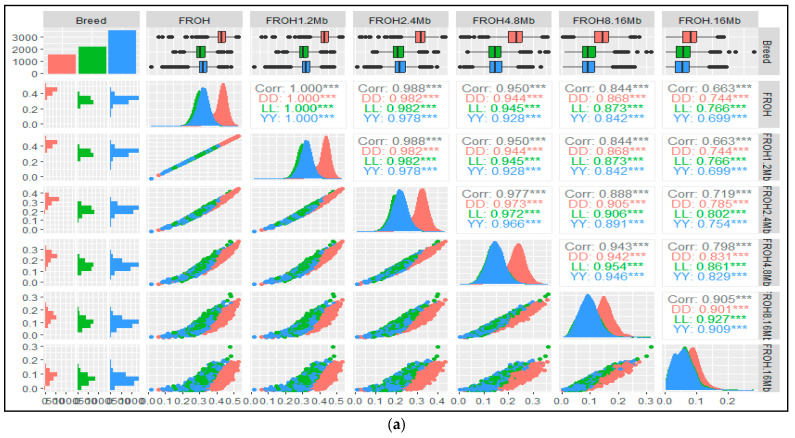

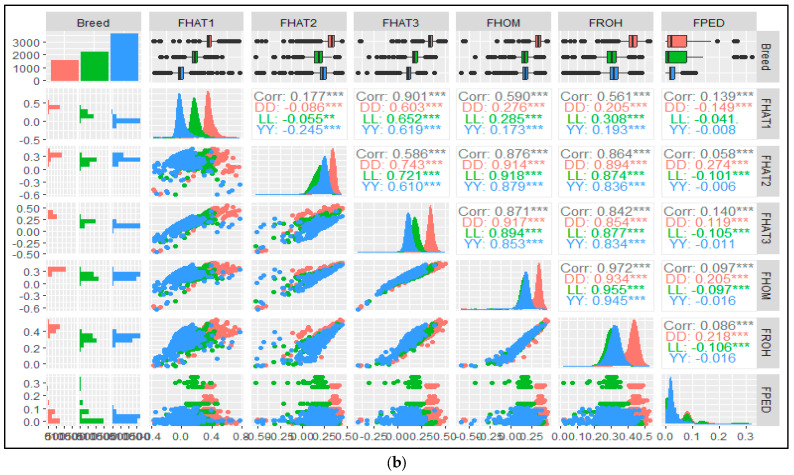
Scatterplots (lower panel) and correlations (upper panel) for three genotypes: (**a**) total genomic inbreeding coefficients (F_ROH_) and by class (F_ROH1–2Mb_, F_ROH2–4Mb_, F_ROH4–8Mb_, F_ROH8–16Mb_, and F_ROH>16Mb_); (**b**) five different genomic inbreeding coefficient estimators and pedigree inbreeding coefficients (F_PED_). F_ROH_: total inbreeding coefficient based on ROH; F_HOM_: inbreeding coefficient based on the observed vs. expected number of homozygous genotypes; F_HAT1_: inbreeding coefficient based on the variance of additive genotype values; F_HAT2_: inbreeding coefficient based on excess homozygosity; F_HAT3_: inbreeding coefficient based upon the correlation between uniting gametes; DD: Duroc; LL: Landrace; and YY: Yorkshire; **: Significant at *p* < 0.01; ***: Significant at *p* < 0.001.

The results in Figure 5a,b present the pairwise Pearson correlations between the total inbreeding coefficients based on the run of homozygosity (F_ROH_) and five ROH classes of genomic inbreeding estimators for the three genotypes. In comparison to F_ROH_, the figure also shows x–y plots depicting the densities of each of the five classes of genomic inbreeding estimators. To better understand the relationship between the inbreeding coefficients obtained using different estimation methods, we performed pairwise comparisons between F_ROH_ and the five classes of F_ROH_ (Figure 5a), F_PED_, and other genomic inbreeding coefficient estimators (Figure 5b). The results demonstrated both positive and negative correlations between genomic and pedigree inbreeding coefficient estimators for the three breeds. Under these conditions, the negative association between the genomic and pedigree inbreeding coefficients indicates that the method used to estimate inbreeding plays a substantial role in determining an individual’s anticipated inbreeding level [57,58]. Consequently, it is essential to thoroughly evaluate the data and results with caution before interpreting and comparing inbreeding coefficients, drawing conclusions based on different inbreeding estimators, and considering the limitations of each technique. The existence of subpopulations within the animals, which are characterized by different allele frequencies from those of the total population, may influence the negative correlation, resulting in inbreeding coefficient variations [23]. This is because one method may provide a positive coefficient to a specific individual, whereas the other results in a negative coefficient, leading to an observed negative correlation.

Although the results in Figure 5a display the pairwise correlations and scatter plots illustrating the relationship between F_ROH_ and the five classes of ROH-based inbreeding coefficients for all three breeds, it reveals a strong absolute correlation of 1.0 between F_ROH_ and F_ROH1–2Mb_ among the observed pairwise correlations for the three breeds. This indicated that the short ROH segment was the primary contributor to the overall F_ROH_ value. Contrary to previous research conducted in a pig population [46], which reported the highest correlation between total F_ROH_ and F_ROH >10Mb_, our findings reveal a different pattern of correlation. Furthermore, we found a strong positive correlation between the inbreeding coefficients obtained through F_ROH2–4Mb_, F_ROH4–8Mb_, F_ROH8–16Mb_, and F_ROH>16Mb_ with F_ROH_. However, as the genomic inbreeding estimator by classes increased from F_ROH2–4Mb_ to F_ROH>16Mb_ for all three breeds, the correlation values gradually decreased (Figure 5a). 

On the other hand, Figure 5b displays scatterplots and Spearman correlations for five additional genomic inbreeding coefficient estimators, along with the pedigree inbreeding coefficient (F_PED_). These estimators included F_ROH_ (based on runs of homozygosity), F_HOM_ (based on the observed versus expected number of homozygous genotypes), F_HAT1_ (based on the variance of additive genotype values), F_HAT2_ (based on excess homozygosity), and F_HAT3_ (based on the correlation between uniting gametes). We observed a negative correlation across the breeds between the estimated inbreeding coefficients from F_HAT1_ and F_HAT2_, as well as between F_HAT1_ and the pedigree inbreeding coefficient (F_PED_). However, except for the Duroc breed, F_PED_ exhibited a negative correlation with F_HAT2_, F_HAT3_, F_HOM_, and F_ROH_. Interestingly, the Yorkshire breeds showed the weakest negative correlation between the F_PED_ and these estimators. We also observed the highest correlation between F_ROH_ and F_HOM_, in line with previous research on pig [46,47,48] and cattle [5,59] populations. The result of a low and negative correlation coefficient between pedigree and genomic inbreeding coefficient estimator shows the effectiveness of using genome-wide SNP information for quantifying the inbreeding coefficient when the pedigree is missing or inaccurate.

## 4. Conclusions

This study examines genome and pedigree based inbreeding coefficients, as well as their correlation, for three Korean commercial breeds reared in five GGP breeding farms in Korea using various estimator models. Between the three breeds, our findings revealed considerable differences in the number, length, and proportions of ROH segments, suggesting varied levels of recent and ancient inbreeding. In all three breeds, shorter segments of 1–2 Mb in length represent the highest proportion of the total ROH. For the shorter genome length classes of ROH, the inbreeding coefficient estimators exhibited relatively high genomic inbreeding coefficients in all three breeds. However, for the longest ROH class, genomic inbreeding coefficient estimators showed lower genomic inbreeding coefficient values based on the ROH class across the three breeds. This pattern indicated that shorter runs of homozygosity contributed to higher inbreeding coefficient values within each breed compared to longer runs of homozygosity, implying that as the size of the runs of homozygosity increases, the average level of genomic inbreeding tends to decrease in all three pig breeds. Our findings revealed a strong correlation between short ROH segments and overall genomic inbreeding values, but a weak to negative correlation between genomic and pedigree-based inbreeding coefficients. This finding revealed homozygosity distribution and inbreeding coefficient estimation for three different commercial pig genotypes based on the ROH generated from SNP markers and pedigree data. These results pave the way for further research into the effects of inbreeding on economically important traits, with the ultimate goal of informing strategies for managing inbreeding and preserving genetic diversity in pig breeding programs.

## Figures and Tables

**Table 1:** Total number of animals used for pedigree and genomic inbreeding coefficient estimation in three breeds.

Breed	Pedigree	F_ROH_	F_HOM_	F_HAT1_	F_HAT2_	F_HAT3_
Duroc	76,987	1586	1586	1586	1586	1586
Landrace	155,628	2256	2556	2256	2256	2256
Yorkshire	409,018	3646	3646	3646	3646	3646

**Table 2:** Descriptive statistics of equivalent complete generations (ECG) of genotyped breeds derived from pedigree data.

Breed	N	Mean	SD	CV	Min	Max	Range	Median
Duroc	76,980	14.89	0.68	4.55	3	15	12	15
Landrace	155,569	14.94	0.44	2.95	4	15	11	15
Yorkshire	408,890	14.92	0.54	3.61	1	15	14	15
All genotype	641,439	14.92	0.54	3.60	1	15	14	15

**Table 3:** Descriptive statistics for the total number, mean, and percentage of five classes of ROH in the three breeds.

ROH_C	Duroc			Landrace			Yorkshire		
	ROH_N	ML(Mb)	P (%)	ROH_N	ML(Mb)	P (%)	ROH_N	ML(Mb)	P (%)
1–2 Mb	462,355	1.09	65.18	576,546	1.072	70.58	983,942	1.085	70.19
2–4 Mb	143,300	2.78	20.20	149,871	2.739	18.35	264,790	2.736	18.89
4–8 Mb	68,029	5.52	9.59	59,050	5.444	7.23	99,388	5.463	7.09
8–16 Mb	24,820	10.87	3.50	20,723	10.789	2.54	35,851	10.855	2.56
>16 Mb	10,880	33.33	1.53	10,708	36.161	1.31	17,810	33.321	1.27
Total (>1)	709,384	53.59	100	816,898	56.205	100	1,401,781	53.461	100

ROH_N: total number count of ROH; ROH: run of homozygosity; ROH_C: run of homozygosity class length, ML: Mean length (Mb).

**Table 4:** The number of ROHs means and percentage coverage per chromosome in three breed populations.

SSC	Duroc			Landrace			Yorkshire		
	ROH_N	ML (Mb)	P (%)	ROH_N	ML (Mb)	P (%)	ROH_N	ML (Mb)	P (%)
1	97,815	2.77	14.31	1,100,18	2.31	14.05	175,188	2.29	13.12
2	41,290	2.81	6.04	47,899	2.48	6.12	80,453	2.50	6.02
3	37,143	2.61	5.43	39,549	2.10	5.05	71,973	2.26	5.39
4	43,602	1.96	6.38	52,394	2.01	6.69	94,402	1.94	7.07
5	37,686	2.13	5.51	42,342	1.74	5.41	75,504	1.87	5.65
6	42,007	2.91	6.15	45,206	2.36	5.77	80,958	2.47	6.06
7	37,957	2.69	5.55	46,366	2.14	5.92	77,746	2.11	5.82
8	33,403	2.40	4.89	41,007	2.58	5.24	65,949	2.48	4.94
9	42,225	2.53	6.18	42,991	2.37	5.49	77,653	2.46	5.81
10	25,340	1.47	3.71	26,857	1.52	3.43	51,211	1.47	3.83
11	25,444	2.06	3.72	28,588	2.00	3.65	52,749	1.85	3.95
12	21,676	1.76	3.17	23,394	1.61	2.99	36,595	1.65	2.74
13	50,978	3.21	7.46	56,999	2.61	7.28	90,905	2.76	6.81
14	39,929	2.89	5.84	46,477	2.32	5.94	92,222	2.10	6.90
15	39,661	2.71	5.80	51,741	2.29	6.61	79,743	2.46	5.97
16	23,152	2.27	3.39	25,944	2.13	3.31	47,058	2.09	3.52
17	24,985	1.60	3.66	33,491	1.44	4.28	49,064	1.42	3.67
18	19,252	1.87	2.82	21,610	1.69	2.76	36,265	1.74	2.72

ROH_N: total number count of ROH; ROH: run of homozygosity; SSC: *Sus scrofa* Chromosomes, ML: Mean length (Mb).

**Table 5:** Genomic inbreeding coefficients based on ROH for five different lengths’ classes and pedigrees in three breeds.

F_ROH_ Class	Duroc			Landrace			Yorkshire		
	*n*	Mean ± SD	CV	*n*	Mean ± SD	CV	*n*	Mean ± SD	CV
F_ROH1–2Mb_	1584	0.42 ± 0.05	12.63	2251	0.30 ± 0.04	13.62	3643	0.32 ± 0.04	13.94
F_ROH2–4Mb_	1582	0.31 ± 0.05	16.12	2251	0.21 ± 0.04	19.8	3643	0.22 ± 0.04	19.44
F_ROH4–8Mb_	1579	0.22 ± 0.05	20.85	2249	0.14 ± 0.04	27.34	3641	0.15 ± 0.04	26.39
F_ROH8–16Mb_	1577	0.14 ± 0.04	29.02	2245	0.09 ± 0.04	38.48	3636	0.09 ± 0.03	36.86
F_ROH > 16Mb_	1567	0.08 ± 0.03	40.79	2212	0.06 ± 0.03	52.14	3587	0.06 ± 0.03	51.32
F_PED_	76,980	0.02 ± 0.03	186.69	155,569	0.01 ± 0.02	178.01	408,891	0.02 ± 0.02	146.43

F_ROH_: Inbreeding coefficient based on ROH classified by length; F_PED:_ Pedigree based inbreeding coefficient.

**Table 6:** Five different genomic and pedigree inbreeding coefficient estimates for three breeds.

Estimator	Duroc			Landrace			Yorkshire		
	*n*	Mean ± SD	CV	*n*	Mean ± SD	CV	*n*	Mean ± SD	CV
F_ROH_	1584	0.42 ± 0.05	12.63	2251	0.30 ± 0.04	13.62	3643	0.32 ± 0.04	13.94
F_HOM_	1586	0.31 ± 0.09	29.28	2256	0.16 ± 0.09	55.49	3646	0.17 ± 0.17	42.17
F_HAT1_	1586	0.50 ± 2.42	485.78	2256	0.28 ± 2.04	718.68	3646	0.04 ± 1.26	3565.36
F_HAT2_	1586	0.23 ± 1.46	631.28	2256	0.09 ± 1.74	1855.5	3646	0.20 ± 0.97	490.98
F_HAT3_	1586	0.36 ± 0.59	162.03	2256	0.19 ± 0.21	113.31	3646	0.12 ± 0.16	141.08
F_PED_	76,980	0.02 ± 0.03	186.69	155,569	0.01 ± 0.02	178.01	408,891	0.02 ± 0.02	146.43

F_ROH_: inbreeding coefficient based on ROH; F_HOM_: based on the observed versus expected number of homozygous genotypes; F_HAT1_: based on the variance of additive genotype values; F_HAT2_: based on excess homozygosity; F_HAT3_: based on the correlation between uniting gametes; F_PED_: pedigree-based inbreeding coefficient.

## Data Availability

The data presented in this study/Supplementary Materials are available on request from the corresponding author due to the privacy of the breeding farm.

## Supplementary Materials

The following supporting information can be downloaded at: https://www.mdpi.com/article/10.3390/ani14172621/s1, Table S1: Descriptive statistics of genomic inbreeding coefficients FROH >1Mb, FROH > 2Mb, FROH > 4Mb, FROH >8Mb, FROH > 16Mb derived from the ROH class across the three breeds; Table S2: Descriptive statistics of genomic inbreeding coefficients of autosomal SSC derived from the runs of homozygosity (ROH) in Duroc pig breeds; Table S3: Descriptive statistics of genomic inbreeding coefficients of autosomal SSC derived from the runs of homozygosity (ROH) in Landrace pig breeds; Table S4: Descriptive statistics of genomic inbreeding coefficients of autosomal SSC derived from the runs of homozygosity (ROH) in Yorkshire pig breeds; Table S5. Summary descriptive statistics of genomic inbreeding coefficients (FROH) across the genomes of the three breeds derived from runs of homozygosity (ROH); Table S6: Summary descriptive statistics of genomic inbreeding coefficients (FHOM) across the genomes of the three breeds derived from observed homozygosity (OHOM) and expected homozygosity (EHOM); Table S7: Summary of descriptive statistics of genomic inbreeding coefficients (FHAT1, FHAT2, FHAT3) across the genomes of the three breeds derived from identity by descent (IBD) regions; Table S8: Summary descriptive statistics of Equivalent Complete Generations (ECG) of the genotyped animals across the three breeds derived from pedigree data; Table S9: Summary descriptive statistics of pedigree inbreeding coefficients (FPED) across the three breeds derived from pedigree data.
