# Supplementary material for: Genomic and Conventional Inbreeding Coefficient Estimation Using Different Estimator Models in Korean Duroc, Landrace, and Yorkshire Breeds Using 70K Porcine SNP BeadChip
Source: Animals (Basel). 2024 Sep 9;14(17):2621. doi: 10.3390/ani14172621 (PMC11394220; doi:10.3390/ani14172621)
Supplement: Supplementary file 1 [file animals-14-02621-s001.zip › animals-3164216-supplementary.pdf]

Supplementary Table

Table S1: Descriptive statistics of genomic inbreeding coefficients $F_{ROH>1Mb}$, $F_{ROH>2Mb}$, $F_{ROH>4Mb}$, $F_{ROH>8Mb}$, $F_{ROH>16Mb}$ derived from the ROH class across the three breeds

Class	N	Mean	SD	CV	Min	Max	Range	Median	Skewness	Kurtosis
Duroc										
FROH1-2 Mb	1584	0.42	0.05	12.63	0.00	0.53	0.53	0.43	-3.46	21.31
FROH2-4 Mb	1582	0.31	0.05	16.12	0.00	0.44	0.43	0.32	-1.87	7.68
FROH4-8 Mb	1579	0.22	0.05	20.85	0.00	0.36	0.35	0.23	-0.95	2.15
FROH8-16 Mb	1577	0.14	0.04	29.02	0.00	0.27	0.27	0.14	-0.30	0.43
FROH>16 Mb	1567	0.08	0.03	40.79	0.01	0.19	0.18	0.08	0.09	-0.08
Landrace										
FROH1-2 Mb	2251	0.30	0.04	13.62	0.00	0.48	0.48	0.30	-0.76	5.19
FROH2-4 Mb	2251	0.21	0.04	19.80	0.00	0.40	0.40	0.21	-0.11	1.68
FROH4-8 Mb	2249	0.14	0.04	27.34	0.00	0.36	0.35	0.14	0.27	1.00
FROH8-16 Mb	2245	0.09	0.04	38.48	0.00	0.31	0.31	0.09	0.48	1.13
FROH>16 Mb	2212	0.06	0.03	52.14	0.01	0.28	0.28	0.06	0.60	1.34
Yorkshire										
FROH1-2 Mb	3643	0.32	0.04	13.94	0.01	0.48	0.47	0.32	-1.40	6.79
FROH2-4 Mb	3643	0.22	0.04	19.44	0.00	0.39	0.39	0.22	-0.55	2.58
FROH4-8 Mb	3641	0.15	0.04	26.39	0.00	0.33	0.33	0.15	0.01	1.08
FROH8-16 Mb	3636	0.09	0.03	36.86	0.00	0.26	0.25	0.09	0.37	0.60
FROH>16 Mb	3587	0.06	0.03	51.32	0.01	0.20	0.19	0.06	0.57	0.46

SD: Standard deviation; CV: coefficient of variation

Table S2: Descriptive statistics of genomic inbreeding coefficients of autosomal SSC derived from the runs of homozygosity (ROH) in Duroc pig breeds

SSC	N	Mean	SD	CV	Min	Max	Range	Median	Skewness	Kurtosis
1	1582	0.54	0.09	17.26	0.01	0.86	0.86	0.54	-0.70	4.08
2	1582	0.45	0.12	26.19	0.00	0.87	0.87	0.44	0.20	0.95
3	1581	0.42	0.11	25.91	0.01	0.85	0.85	0.41	0.28	0.86
4	1581	0.38	0.11	29.56	0.01	0.95	0.94	0.37	0.44	1.23
5	1581	0.46	0.11	23.52	0.01	0.89	0.89	0.46	-0.05	0.95
6	1580	0.45	0.09	19.94	0.00	0.78	0.78	0.46	-0.43	2.17
7	1582	0.48	0.11	23.99	0.01	0.95	0.94	0.48	-0.19	0.81
8	1580	0.34	0.13	36.58	0.00	0.92	0.92	0.32	0.60	0.54
9	1581	0.44	0.10	22.42	0.00	0.89	0.88	0.43	0.21	1.79
10	1578	0.30	0.10	35.35	0.01	0.81	0.80	0.29	0.72	1.32
11	1579	0.38	0.12	32.68	0.01	0.87	0.86	0.37	0.38	0.55
12	1578	0.35	0.11	31.15	0.01	0.80	0.79	0.35	0.38	0.38
13	1582	0.47	0.13	27.85	0.00	0.93	0.92	0.47	0.14	0.53
14	1581	0.47	0.11	24.22	0.01	0.88	0.87	0.47	0.07	0.96
15	1581	0.40	0.11	26.62	0.00	0.86	0.85	0.39	0.31	1.05
16	1580	0.38	0.13	33.75	0.01	0.92	0.91	0.37	0.45	0.71
17	1579	0.36	0.11	30.06	0.01	0.88	0.88	0.35	0.68	1.67
18	1576	0.37	0.13	34.69	0.02	1.00	0.98	0.35	0.88	1.37

SSC: *Sus scrofa* chromosomes; N: number of animals; SD: Standard deviation; CV: coefficient of variation

Table S3: Descriptive statistics of genomic inbreeding coefficients of autosomal SSC derived from the runs of homozygosity (ROH) in Landrace pig breeds

SSC	N	Mean	SD	CV	Min	Max	Range	Median	Skewness	Kurtosis
1	2250	0.36	0.11	29.78	0.03	0.89	0.86	0.34	0.89	1.38
2	2250	0.33	0.13	40.18	0.01	0.88	0.87	0.30	0.88	0.83
3	2249	0.26	0.12	46.64	0.01	0.82	0.81	0.23	1.15	1.65
4	2250	0.33	0.12	36.69	0.03	0.92	0.89	0.31	0.93	1.35
5	2249	0.29	0.11	36.25	0.01	0.89	0.89	0.28	0.93	1.76
6	2250	0.28	0.10	34.27	0.00	0.79	0.79	0.27	0.94	1.89
7	2250	0.33	0.11	32.68	0.01	0.92	0.91	0.31	1.10	2.28
8	2249	0.32	0.13	40.67	0.02	0.91	0.88	0.31	0.73	1.02
9	2249	0.29	0.11	37.19	0.01	0.87	0.85	0.28	0.87	1.63
10	2246	0.23	0.11	49.72	0.01	0.85	0.84	0.21	1.08	2.07
11	2249	0.29	0.12	41.25	0.01	0.87	0.87	0.28	0.86	1.18
12	2246	0.25	0.12	48.21	0.01	0.79	0.78	0.23	0.85	1.09
13	2251	0.30	0.12	40.57	0.00	0.87	0.86	0.28	1.12	2.06
14	2250	0.31	0.12	38.36	0.02	0.90	0.88	0.30	1.05	2.11
15	2251	0.31	0.11	35.91	0.00	0.82	0.81	0.29	0.92	1.45
16	2249	0.28	0.13	46.71	0.01	0.96	0.95	0.26	1.09	2.13
17	2248	0.31	0.12	38.68	0.00	0.96	0.96	0.29	0.95	1.97
18	2249	0.26	0.14	51.36	0.01	0.91	0.90	0.24	1.22	1.95

SSC: *Sus scrofa* chromosomes; N: number of animals; SD: Standard deviation; CV: coefficient of variation

Table S4: Descriptive statistics of genomic inbreeding coefficients of autosomal SSC derived from the runs of homozygosity (ROH) in Yorkshire pig breeds

SSC	N	Mean	SD	CV	Min	Max	Range	Median	Skewness	Kurtosis
1	3643	0.35	0.10	29.28	0.00	0.95	0.94	0.34	0.72	1.46
2	3643	0.34	0.13	36.82	0.01	0.86	0.85	0.33	0.45	0.22
3	3642	0.31	0.11	36.74	0.01	0.93	0.92	0.29	0.87	1.94
4	3643	0.35	0.11	31.54	0.00	0.89	0.89	0.34	0.65	1.24
5	3641	0.35	0.12	33.70	0.01	0.87	0.86	0.34	0.56	0.53
6	3641	0.32	0.10	31.13	0.00	0.82	0.82	0.31	0.66	1.22
7	3643	0.33	0.11	31.75	0.00	0.94	0.94	0.32	1.01	2.33
8	3642	0.30	0.13	42.79	0.00	0.97	0.97	0.28	0.89	1.07
9	3642	0.34	0.11	33.18	0.01	0.88	0.87	0.33	0.59	1.03
10	3636	0.26	0.11	40.60	0.00	0.84	0.84	0.25	0.78	1.23
11	3640	0.31	0.13	40.90	0.01	0.87	0.86	0.29	0.82	1.26
12	3635	0.24	0.11	43.55	0.01	0.86	0.85	0.23	0.83	1.55
13	3641	0.32	0.13	42.10	0.00	0.97	0.97	0.29	1.18	2.34
14	3642	0.35	0.11	32.88	0.01	0.89	0.89	0.33	0.78	1.59
15	3642	0.32	0.12	38.01	0.00	0.87	0.86	0.30	0.80	1.09
16	3640	0.31	0.13	40.62	0.01	0.92	0.91	0.30	0.70	1.08
17	3640	0.27	0.12	44.38	0.01	0.87	0.87	0.26	0.76	0.88
18	3639	0.28	0.13	47.54	0.01	1.00	0.99	0.26	1.15	2.10

SSC: Sus scrofa chromosomes; N: number of animals; SD: Standard deviation; CV: coefficient of variation

Table S5. Summary descriptive statistics of genomic inbreeding coefficients (F_{ROH}) across the genomes of the three breeds derived from runs of homozygosity (ROH).

Breed	N	Mean	SD	CV	Min	Max	Range	Median	Skewness	Kurtosis
DD	1584	0.42	0.05	12.63	0.00	0.53	0.53	0.43	-3.46	21.31
LL	2251	0.30	0.04	13.62	0.00	0.48	0.48	0.30	-0.76	5.19
YY	3643	0.32	0.04	13.94	0.01	0.48	0.47	0.32	-1.40	6.79

Table S6: Summary descriptive statistics of genomic inbreeding coefficients (F_{HOM}) across the genomes of the three breeds derived from observed homozygosity (O_{HOM}) and expected homozygosity (E_{HOM})

Breed	N	Mean	SD	CV	Min	Max	Range	Median	Skewness	Kurtosis
DD	1586	0.31	0.09	28.98	-1.36	0.44	1.79	0.33	-9.74	144.20
LL	2256	0.16	0.09	55.49	-1.69	0.50	2.19	0.16	-12.22	234.72
YY	3646	0.17	0.07	42.17	-1.60	0.40	2.00	0.18	-8.94	176.04

Table S7: Summary of descriptive statistics of genomic inbreeding coefficients (F_{HAT1}, F_{HAT2}, F_{HAT3}) across the genomes of the three breeds derived from identity by descent (IBD) regions.

Estimator	N	Mean	SD	CV	Min	Max	Range	Median	Skewness	Kurtosis
**Duroc**										
F_{HAT1}	1586	0.50	2.42	485.78	-0.37	58.49	58.86	0.36	22.14	500.55
F_{HAT2}	1586	0.23	1.46	631.28	-39.50	0.45	39.96	0.33	-24.20	624.66
F_{HAT3}	1586	0.36	0.59	162.03	-0.40	20.72	21.12	0.34	29.22	939.20
**Landrace**										
F_{HAT1}	2256	0.28	2.04	718.68	-0.28	50.30	50.58	0.18	21.37	474.78
F_{HAT2}	2256	0.09	1.74	1855.50	-43.22	0.42	43.64	0.19	-22.10	511.21
F_{HAT3}	2256	0.19	0.21	113.31	-0.30	8.60	8.90	0.18	31.27	1136.25
**Yorkshire**										
F_{HAT1}	3646	0.04	1.26	3565.36	-0.36	54.32	54.68	-0.02	36.71	1415.40
F_{HAT2}	3646	0.20	0.97	490.98	-42.79	0.45	43.24	0.24	-36.20	1404.52
F_{HAT3}	3646	0.12	0.16	141.08	-0.42	7.60	8.01	0.11	37.43	1568.56

Table S8: Summary descriptive statistics of Equivalent Complete Generations (ECG) of the genotyped animals across the three breeds derived from pedigree data

Breed	N	Mean	SD	CV	Min	Max	Range	Median	Skewness	Kurtosis
DD	76980	14.89	0.68	4.55	3	15	12	15	-9.54	110.67
LL	155569	14.94	0.44	2.95	4	15	11	15	-11.44	157.4
YY	408890	14.92	0.54	3.61	1	15	14	15	-11.39	168.79
All	641439	14.92	0.54	3.60	1	15	14	15	-11.24	161.58

Table S9: Summary descriptive statistics of pedigree inbreeding coefficients (F_{PED}) across the three breeds derived from pedigree data

Breed	N	Mean	SD	CV	Min	Max	Range	Median	Skewness	Kurtosis
DD	76980	0.02	0.03	186.69	0.00	0.30	0.30	0.00	3.81	20.95
LL	155569	0.01	0.02	178.01	0.00	0.25	0.25	0.00	2.97	12.54
YY	408891	0.02	0.02	146.43	-0.01	0.32	0.33	0.01	3.08	18.10
